# First all-in-one diagnostic tool for DNA intelligence: genome-wide inference of biogeographic ancestry, appearance, relatedness, and sex with the Identitas v1 Forensic Chip

**DOI:** 10.1007/s00414-012-0788-1

**Published:** 2012-11-13

**Authors:** Brendan Keating, Aruna T. Bansal, Susan Walsh, Jonathan Millman, Jonathan Newman, Kenneth Kidd, Bruce Budowle, Arthur Eisenberg, Joseph Donfack, Paolo Gasparini, Zoran Budimlija, Anjali K. Henders, Hareesh Chandrupatla, David L. Duffy, Scott D. Gordon, Pirro Hysi, Fan Liu, Sarah E. Medland, Laurence Rubin, Nicholas G. Martin, Timothy D. Spector, Manfred Kayser, on behalf of the International Visible Trait Genetics (VisiGen) Consortium

**Affiliations:** 1 The University of Pennsylvania, Office 1016, Abramson Building, 3615 Civic Center Bvld., Philadelphia, PA 19104-4399 USA; 2 Identitas Inc., 1115 Broadway, 12th Floor, New York, NY 10010 USA; 3 Department of Forensic Molecular Biology, Erasmus MC University Medical Center Rotterdam, PO Box 2040, 3000 CA Rotterdam, The Netherlands; 4 Centre of Forensic Sciences, 25 Grosvenor Street, Toronto, ON M7A 2G8 Canada; 5 Yale University School of Medicine, PO Box 208005, New Haven, CT 06520-8005 USA; 6 Institute of Applied Genetics, Department of Forensic and Investigative Genetics, University North Texas Health Science Center, 3500 Camp Bowie Blvd, Fort Worth, TX 76107 USA; 7 Laboratory Division, Federal Bureau of Investigation, 2501 Investigation Parkway, Quantico, VA 22135 USA; 8 Institute for Maternal and Child Health, IRCCS Burlo Garofolo, University of Trieste, Piazzale Europa1, 34127 Trieste, Italy; 9 New York City Office of Chief Medical Examiner, 421 East 26th Street, New York, NY 10016 USA; 10 Queensland Institute of Medical Research, Royal Brisbane Hospital, Locked Bag 2000, Herston, Brisbane, Queensland 4029 Australia; 11 Anjin Solutions, 34 Downing Lane, Voorhees, NJ 08043 USA; 12 Department of Twin Research, King’s College London, St. Thomas’ Hospital, Westminster Bridge Road, London, SE1 7EH UK

**Keywords:** DNA intelligence, Forensic DNA phenotyping, SNP, Prediction, Relatedness, Kinship, Ancestry, Eye color, Hair color, Sex

## Abstract

When a forensic DNA sample cannot be associated directly with a previously genotyped reference sample by standard short tandem repeat profiling, the investigation required for identifying perpetrators, victims, or missing persons can be both costly and time consuming. Here, we describe the outcome of a collaborative study using the Identitas Version 1 (v1) Forensic Chip, the first commercially available all-in-one tool dedicated to the concept of developing intelligence leads based on DNA. The chip allows parallel interrogation of 201,173 genome-wide autosomal, X-chromosomal, Y-chromosomal, and mitochondrial single nucleotide polymorphisms for inference of biogeographic ancestry, appearance, relatedness, and sex. The first assessment of the chip’s performance was carried out on 3,196 blinded DNA samples of varying quantities and qualities, covering a wide range of biogeographic origin and eye/hair coloration as well as variation in relatedness and sex. Overall, 95 % of the samples (*N* = 3,034) passed quality checks with an overall genotype call rate >90 % on variable numbers of available recorded trait information. Predictions of sex, direct match, and first to third degree relatedness were highly accurate. Chip-based predictions of biparental continental ancestry were on average ~94 % correct (further support provided by separately inferred patrilineal and matrilineal ancestry). Predictions of eye color were 85 % correct for brown and 70 % correct for blue eyes, and predictions of hair color were 72 % for brown, 63 % for blond, 58 % for black, and 48 % for red hair. From the 5 % of samples (*N* = 162) with <90 % call rate, 56 % yielded correct continental ancestry predictions while 7 % yielded sufficient genotypes to allow hair and eye color prediction. Our results demonstrate that the Identitas v1 Forensic Chip holds great promise for a wide range of applications including criminal investigations, missing person investigations, and for national security purposes.

**Electronic supplementary material:**

The online version of this article (doi:10.1007/s00414-012-0788-1) contains supplementary material, which is available to authorized users.

## Introduction

There have been, and likely will continue to be, forensic cases where the evidentiary DNA profile does not directly match that of a known individual or any reference sample profile contained within a national DNA database. In addition, current forensic DNA profiling has provided, and likely will continue to provide, little or no information in a number of missing person cases, including mass disaster identification, where scant information is available on the putative identity of the remains found. Traditional policing places heavy reliance on human eyewitnesses to enable investigators to identify suspects. While eyewitness reports have been shown to be helpful, they are highly error prone [1, 2], and consequently a number of people convicted on the basis of eyewitness identification evidence have been exonerated through forensic DNA testing [2]. The emerging field of DNA intelligence allows novel investigative leads to be developed directly from the DNA of a forensic sample that can help in identifying persons previously unknown to the authorities. This has the benefit of reducing reliance on human eyewitness accounts and can provide leads in the many cases without known human eyewitnesses [3, 4]. Valuable information in this respect includes biogeographic ancestry and externally visible characteristics (EVC) of the unknown sample donor such as sex, eye color and hair color, and others, via the discipline of Forensic DNA Phenotyping (FDP), as well as the relatedness of the unknown donor with alleged family members.

Worldwide scientific initiatives such as the Human Genome Project [5, 6] and subsequently the International HapMap Project [7–10] together have laid the foundations for the discovery and large-scale population diversity catalogues of several millions of single nucleotide polymorphisms (SNPs). Highly effective massively parallel SNP genotyping platforms using microarray technology were developed from these resources allowing genome-wide analysis of currently over a million SNPs in a single test [11, 12]. High-resolution SNP microarrays have been used in various human population studies, such as in the worldwide Human Genome Diversity Panel (HGDP-CEPH) [13, 14], and in population studies within continents such as Europe [15], Asia [16], Africa [17], India [18], and Oceania [19]. Together with the HapMap data resources [10] and candidate marker studies including those on Y-chromosomal and mitochondrial DNA diversity [20], substantial knowledge on DNA-based inference of biogeographic ancestry has begun to be realized. From these datasets, so-called ancestry-informative DNA markers (AIMs) have been developed. Autosomal AIM sets usually provide ancestry resolution at the level of broad geographic regions such as continents [21–25], whereas with some particular Y-chromosomal and mitochondrial AIMs, within-continental resolution can be achieved [4, 20]. In some efforts, multiplex AIM panels suitable for forensic applications have been developed for autosomal, Y-chromosomal, and mitochondrial SNPs [26–32]. However, especially when it comes to recombining autosomal markers, such reduced AIM panels tend to provide less ancestry resolution than high-resolution SNP microarrays [4].

Moreover, high-resolution SNP microarrays have been used in genome-wide association studies (GWASs) to discover SNPs involved in human EVCs, most notably eye and hair color [33–37]. From these studies, and from candidate gene studies [38–42], DNA markers predictive for human eye and hair color categories have been identified [37, 43–48]. The first multiplex tools for DNA-based eye and/or hair color prediction have been made available recently [49–53] of which at least one, the IrisPlex system for blue and brown eye color prediction [49], has already been successfully validated for forensic applications [54].

All conventional efforts to develop diagnostic tools for DNA intelligence however were restricted to specific elements and were analyzed separately. This limited approach is not only partly due to the incremental nature of the research but also because of technological restrictions on the number of SNPs that could be genotyped reliably in single multiplex assays, suitable for forensic samples. For instance, the SNaPshot chemistry, which is the most widespread SNP typing technology in the forensic genetic field, only allows for the analysis of up to a few dozen SNPs in a single multiplex assay. Thus, several separate DNA tests needed to be performed to enable screening of sufficient markers for comprehensive DNA intelligence efforts. In many forensic cases, however, input DNA amounts are substantially limited, restricting the number of independent DNA tests that can be performed. A single multiplex SNP typing tool including a large number of SNPs is therefore needed that allows various elements of DNA intelligence to be inferred, in parallel, from a single forensic DNA aliquot.

In an international, industry–academic collaboration, the International Visible Trait Genetic (VisiGen) Consortium, the Identitas Version 1 (v1) Forensic Chip was developed. The chip, based on well-established Illumina Infinium technology, allows simultaneous genotyping of 192,658 autosomal SNPs of genome-wide distribution, 3,012 Y-chromosomal, 5,075 X-/XY-chromosomal, and 428 mitochondrial SNPs. The genome-wide SNPs were selected primarily for kinship and biogeographic ancestry inference. The panel though was enriched with SNPs that were previously established to have predictive value for biogeographic ancestry and several appearance traits most notably eye and hair color. Herein, the first performance study of the Identitas v1 Forensic Chip is reported based both on data established by consortium members and data from governmental forensic labs in the USA and Canada. A total of 3,196 DNA samples collected from around the world were analyzed. Many of them have recorded sex, continental ancestry, and eye and hair color information, and, in some cases, details of relatedness. The DNA samples were of varying quality and quantity as a result of titration and degradation experiments, and the establishment of mock case-work samples. Genotype quality was assessed, and predictions of sex, biogeographic ancestry, hair color, eye color, and kinship were derived and compared with study-recorded trait data, where available. This study provides the first insights into the performance and feasibility of the Identitas v1 Forensic Chip, the first all-in-one diagnostic tool dedicated to DNA intelligence.

## Material and methods

### DNA samples and available individual information

A total of 3,196 DNA samples were studied. In part, samples were deliberately drawn from a highly biogeographically diverse set of individuals, in order to investigate the quality of prediction of biogeographic ancestry. For a subset of 2,780 individuals, self-reported or site-reported ancestry information was available. For the purposes of obtaining accuracy estimates, individuals were categorized into five major biogeographic groups, plus another category that included, for example, West Asians and individuals from Oceania, for whom HapMap v3 reference samples were not available. The breakdown of site-reported ancestry, where available, and without masking the inevitable overlap in categorizations, was as follows (count in parentheses)—1,880 individuals were categorized as European descent including the following self-declared or site-reported groups: Adygei (25), Austrian (2), Azerbaijani (38), British (682), Caucasian (46), Chuvash (25), European (380), Georgian (117), German (2), Hungarian (25), Irish (303), Italian (2), Komi (25), Poland (4), and White (204) and 240 individuals were categorized as East Asian descent including the following self-declared or site-reported groups: Ami (25), Asian (20), Atayal (25), Cambodian (20), Chinese (1), Hakka (25), Japanese (25), Korean (24), Laotian (25), Micronesian (25), and Yakut (25). Although Micronesia is in the Western Pacific, Micronesians genetically are known to be largely of East Asian ancestry as result of their migration history (although a Near Oceanian ancestry component exists as well) [55], which justifies their grouping here; 176 individuals were categorized as African descent including the following self-declared or site-reported groups: African (2), African American (3), Barbadian (2), Black (108), Ethiopian (25), Hausa (25), and Jamaican (11); 123 individuals were categorized as South American descent including the following self-declared or site-reported groups: Karitiana (25), Mayan (25), Pima (25), Quechua (23), and Ticuna (25); 31 individuals were categorized as South Asian descent including the following self-declared or site-reported groups: Kerala (25) and South Asian (6); and 330 were categorized outside of these major biogeographic groups: Druze (29), Gimi (15), Inuit (19), Iraq (2), Kazakhstani (57), Khanty (25), Lebanese (8), Mixed Race (34), Nasioi (17), Other (21), Uzbek (78), and Yemeni (25).

The majority of the aforementioned DNA samples came from TwinsUK and the QIMR Twin Registry studies, as well as from the Yale collection as described in detail elsewhere [56–58]. For those samples, DNA was derived from whole blood either directly or from blood-derived lymphoblastoid cell lines. Additionally, a subset of 171 DNA samples was derived from more forensically relevant sources which included (counts in parentheses): hair (2), buccal swab (97), blood swab (9), semen (2), vaginal swab (3), saliva (3), mucus (1), gum (3), drink container swab (8), cigarette butt (10), chap-stick swab (1), swab of tape ends (1), saliva/blood mixture (1), and vaginal swab/semen mixtures (30). Two groups contributed sexual-assault type samples. Twenty-nine samples, consisting of either vaginal or buccal samples from female donors (*N* = 8), were spiked with varying amounts of semen from male donors (*N* = 3) and subjected to a standard differential extraction procedure. Results derived using Plexor HY (Promega), a dual autosomal and male-specific quantification assay, indicated that the percentage of male DNA ranged from 100 % to none. One group submitted an additional mixture made up of vaginal swab DNA plus semen.

Sensitivity samples were contributed by two groups. From the first group, four samples were run in a dilution series of total DNA input at 200, 100, 50, 10, 3.3, and 1 ng, leading to a total of 24 samples. The DNA concentration per sample was measured twice using the Quantifiler Human DNA quantification kit (Applied Biosystems) to determine total concentrations of 40 ng/μl down to 200 pg/μl, using a 5-μl sample volume. The second group applied serial dilution to five reference sample extracts of 500 ng to 50 pg total DNA, yielding total DNA concentrations for each sample of 25, 2.5, 0.25, 0.025, and 0.0025 ng/μl. Measurements were taken using Plexor HY (Promega).

Degraded samples were experimentally derived from four pre-extracted DNA samples using (a) titrated DNase treatments and (b) by subjecting samples to ultraviolet (UV) light time courses. Quantified dilutions at concentrations of 20 and 2 ng/μl, in a total sample volume of 110 μl (Quantifiler Human DNA quantification kit, Life Technologies), underwent DNase (Sigma) treatment (0.1 U on each 110-μl volume) for 0, 1, 5, and 10 min with approximately 10 μl of each sample extracted. Thus, three time points and two different concentrations were assessed, for each of the four DNA samples (24 samples altogether). Samples were exposed to UV light for time intervals of 0, 5, 10, and 30 min, using the Bio-Link (Vilber Lourmat) at a strength of 50 J/cm^2^. Three time points for two different concentrations (100 and 10 ng total DNA per 5 μl) were derived for each of four samples (24 samples altogether).

Prior to chip genotyping, DNA samples collated from the different collaborator sites were re-quantified using a PicoGreen-based assay (Life Technologies). Genotyping followed the standard Illumina Infinium iSelect protocol (www.illumina.com). Fluorescence intensities were detected by the Illumina iScan and analyzed using Illumina’s BeadStudio software. The reaction volumes were 2 μl for quality checking and 5 μl for genotyping; additional volume was required to allow for pipetting. In the data received from Illumina, samples were categorized as either “passed” or “failed” using the standard control metrics from Illumina, with failure assigned to samples with more than 10 % missing genotypes calls.

### Statistical analyses

Female-derived DNA was inferred on the basis of X-chromosome heterozygosity; the presence of Y-chromosome genotypes confirmed the presence of male-derived DNA. Biparental biogeographic ancestry predictions were conducted using a marker subset of 81,031 autosomal SNPs exhibiting low correlation (low linkage disequilibrium). Patrilineal ancestry was derived from a subset of 484 Y-chromosomal SNPs, and matrilineal ancestry was derived using a subset of 280 mitochondrial SNPs, for which phylogenetic and geographic origin information was available [59–61]. Principal components analysis (PCA) was conducted using, as a reference, data from HapMap version 3 [10]. Individuals were assigned to continental groups using a simple distance-based clustering algorithm. Model-based clustering, assuming five populations with distinct allele frequencies, was conducted using the same reference set [62, 63]. Hair color and eye color were predicted using multinomial logistic regression models of predictive SNPs as described elsewhere [49, 52] with the difference that the following four hair color-predicting DNA variants from the *MC1R* gene could not be implemented on the chip: N29insA (INDEL), Y152OCH, rs1805007, and rs1805009; the hair color prediction model used was adjusted accordingly. Degrees of relatedness were inferred for each pair by calculation of the proportion of the genome shared identical by state (IBS) based upon 192,576 autosomal markers with minor allele frequencies greater than 1 %, and less than 5 % missing genotypes [64].

## Results

### Technical chip performance with relevance for phenotype inference

#### Genotype reproducibility

Concordance testing using different SNP microarray platforms was performed on 102 QIMR samples genotyped in this study on the Identitas v1 Forensic Chip and previously, at a different laboratory, on the Infinium 610-Q (Illumina) GWAS arrays [65]. Up to 107,262 SNPs directly overlap between both arrays. The observed discordance was 92 out of the total of 10,831,289 genotype calls (rate 0.00085 %, data available on request). Although, these data do not show which of the two microarrays produced the *bona fide* genotype, the high concordance rate of >99.999 % indicates that the reproducibility of genotypes from the Identitas v1 Forensic Chip, at least for samples of similar quality and quantity as the 102 tested here, is very high.

#### Sensitivity testing

Serial dilutions were conducted by one group for five DNA samples extracted from buccal samples of five individuals of European biogeographic origin. Each was diluted to contain 175, 17.5, 1.75, 0.175, or 0.0175 ng in the 7-μl reaction volume for chip genotyping. For the lowest concentration of 0.0175 ng reaction DNA, all five samples failed platform QC with overall genotype call rate <90 %, none had the full complement of markers for hair and eye color prediction, and only one provided an accurate prediction of biogeographic ancestry (quantitative method, see later). At the next level of DNA concentration, 0.175 ng reaction DNA, one sample passed platform QC, one had the full complement of markers for hair and eye color prediction, and three provided accurate predictions of biogeographic ancestry. At the next level, 1.75 ng reaction DNA, three passed platform QC, three had the full complement of markers for hair and eye color prediction, and all five provided accurate predictions of biogeographic ancestry. At higher concentrations, amounting to 17.5 and 175 ng total DNA, 9/10 passed platform QC, 8/10 had the full complement of markers for hair and eye color prediction, and all 10 provided accurate predictions of biogeographic ancestry.

Serial dilutions were conducted by a second group for four DNA samples extracted from blood of four individuals of European biogeographic origin. Each was diluted to 200, 100, 50, 10, 3.3, and 1 ng in a 5-μl volume. It is noted that for this set, further dilution was required to increase volume prior to genotyping, so the amount of DNA used may be lower. For samples at the lowest concentration (1 ng reaction DNA), all failed platform QC, none had the full complement of markers for hair and eye color prediction, but all four provided an accurate prediction of biogeographic ancestry. At the next level of 3.3 ng reaction DNA, one passed QC, one had the full complement of markers for hair and eye color prediction, and all four provided accurate predictions of biogeographic ancestry. At the higher concentrations of 10, 50, and 100 ng total DNA, all samples passed QC, all had the full complement of markers for hair and eye color, and all provided accurate predictions of biogeographic ancestry.

Despite the preliminary character of the sensitivity testing performed here with small sample sizes, these results demonstrate that biogeographic ancestry may be accurately predicted from as little as 1.75 ng DNA or even, in some cases, as little as 0.175 ng DNA. For other traits, predictive success is dependent on generating sufficient relevant genotypes.

#### Degradation testing

Twenty-four samples derived from four initial DNA samples were subjected to severe ultraviolet degradation. Upon genotyping, only three passed platform quality checks. They corresponded to three of the 100 ng samples at the first time point, and all had accurate predictions of biogeographic ancestry and sufficient genotypes to allow hair and eye color prediction. The remaining 21 samples had between 18 and 50 % missing genotypes, none had sufficient genotypes to allow hair and eye color prediction, but four (all 10 ng at first time point) provided accurate predictions of biogeographic ancestry.

The same 24 samples were subjected to less severe, enzymatic degradation. Median concentration for the set was <1 ng/μl. Upon genotyping, five samples failed platform quality checks: one at a degradation time of 1 min, two at 5 min, and three at the final time point of 10 min. The five samples had between 11 and 21 % missing genotypes, compared to less than 10 % missing genotypes in the 19 samples which passed platform quality checks. The elevated failure rate in this small degradation subset confirms the damaging effect of nucleases on DNA genotyping performance. A total of 20/24 samples however had sufficient genotypes to allow prediction of hair color and eye color, and all 24 enzymatically degraded samples led to accurate estimates of biogeographic ancestry.

#### Analysis of forensic-type samples

A total of 141 single-source DNA samples extracted from hair, buccal swab, blood swab, semen, vaginal swab, saliva, mucus, gum, drink container swab, cigarette butt, chap-stick swab, and swab of tape ends were examined, to assess performance in more typical case-work samples. DNA concentration was measured by PicoGreen and found to be generally low (median = 2.7 ng/μl). Of these 141 samples, 102 passed QC and had median PicoGreen-based concentration of 3.1 ng/μl (range 0.7–56.6 ng/μl). A total of 102/141 (72 %) samples had sufficient genotypes to allow prediction of hair color and eye color. Biogeographic ancestry was available for 125 of the samples, the majority (85 %) of whom were of European ancestry. A total of 110 out of 125 (88 %) of the predictions were correct on the level of continental ancestry. There were no clear differences in performance among sample types; correct predictions were obtained from blood swab, buccal swab, semen, vaginal swab, mucus, gum, drink container swab, and hair.

In addition, a total of 30 multisource DNA samples from simulated sexual assault material extracted after differential lysis were examined. Again, DNA concentration tended to be low (median = 1.6 ng/μl, range 0.8–61.4 ng/μl). A total of 19/30 samples passed quality checks; however, upon unblinding at source, it emerged that only 13 of them contained male DNA. For all 13 samples, the Y-chromosome haplogroup was obtained and their assumed geographic region of origin was found to be consistent with site-reported biogeographic ancestry.

### Chip-based inference of sex, ancestry, appearance, and relatedness

Across the whole study, a total of 3,034 (95 %) samples passed platform quality checks with overall genotype call rates >90 %, while 162 samples (5 %) failed this threshold. In the following sections, results are presented of the analysis of the 3,034 DNA samples that passed quality control checks.

#### Inference of sex

A two-pronged approach was taken for chip-based prediction of whether the DNA used was derived from a man or a woman. Y-chromosome haplogroups, derived from known non-recombining male-specific SNPs, were obtained for 1,114 DNA samples, indicating that they were derived from males. In addition, X-chromosome heterozygosity was determined for all samples on the basis of 5,066 X-chromosome-specific markers (without a homologue on the Y chromosome), with an estimated mean heterozygosity of <0.2 implying male-derived DNA and an estimated mean heterozygosity of >0.8 implying female-derived DNA [64]. Intermediate values were considered inconclusive. Twelve conflicts, between chip-predicted and site-reported sex information, were obtained in the 1,588 samples with site-reported sex information available. Four samples were predicted to be derived from males on the basis of both identified Y-haplogroup and low X-chromosome heterozygosity but were unblinded as being derived from females from the records. Seven samples were predicted to be derived from females on the basis of no inferable Y-haplogroup and high X-chromosome heterozygosity but were unblinded as being derived from males from records. Further nongenetic sex data could not be obtained for these 11 individuals. A plausible explanation however is that DNA mix-ups at some stage may have occurred for these samples, which would correspond to a male–female sample mix-up rate of 0.69 % in our study. One of the 1,588 samples, unblinded as female, was wrongly predicted to be male on the basis of low X-chromosome heterozygosity alone but had no Y-chromosome haplogroup inferable. This sample would have represented a sex misclassification based solely on the X-chromosome heterozygosity approach, emphasizing the importance of using data from both X- and Y-chromosome SNPs for inferring sex from these chip data.

#### Inference of continental biogeographic ancestry

**Fig. 1:**
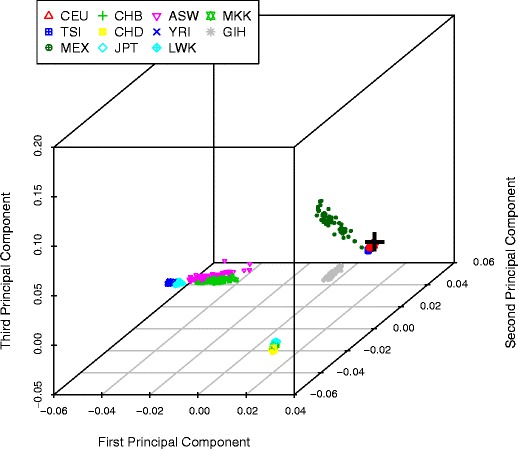
Three-dimensional principal component analysis plot for a DNA sample from a European individual denoted by a *black cross*, together with reference data from HapMap v3 i.e., from individuals of European descent (*CEU* Utah residents with Northern and Western European ancestry from the CEPH collection; *TSI* Tuscans in Italy), African descent (*ASW* African ancestry in Southwest USA; *LWK* Luhya in Webuye, Kenya; *MKK* Maasai in Kinyawa, Kenya; *YRI* Yoruba in Ibadan, Nigeria), East Asian descent (*CHB* Han Chinese in Beijing, China; *CHD* Chinese in Metropolitan Denver, CO; *JPT* Japanese in Tokyo, Japan), South Asian descent (*GIH* Gujarati Indians in Houston, TX), and South American descent (*MEX* Mexican ancestry in Los Angeles, CA)

A three-dimensional PCA plot of the HapMap 3 reference data [10] led to the clustering of individual samples into five, somewhat separated, main descent groups: European descent (CEU, TSI), African descent (ASW, LWK, MKK, YRI), East Asian descent (CHB, CHD, JPT), South Asian descent (GIH), and South American descent (MEX) (Fig. 1). This reference dataset was then used to classify the study samples according to their biparental continental biogeographic ancestry via a distance-based clustering algorithm. For example, the black cross in Fig. 1 represents an individual sample, unblinded as “White” classified by self-reported ancestry information, which appears very close the European-descent HapMap reference samples (CEU and TSI), highlighting the most likely European biparental genetic ancestry of this individual. Self- or site-reported biogeographic ancestry on the continental level was available for 2,688 out of 3,034 samples. Overall, PCA clustering led to good prediction accuracy for certain categories of biparental genetic ancestry. Predictions of European ancestry were 97 % consistent with self-declared or site-reported ancestry, predictions of African ancestry were 88 % consistent with self/site report, and predictions of East Asian ancestry were 97 % consistent with self/site report. Predictions of South Asian ancestry, however, were lower (69 % consistent with self/site report), while predictions of South American ancestry were poor. Specifically 39 % (52/134) of the predictions of South American ancestry were self-/site-categorized outside of the main five major biogeographic ancestry groups (they were 19 Khanty, 9 mixed race, 9 Other, 1 Lebanese, 1 Kazakhstan, 5 Uzbekistan, and 8 Inuit Aborigine).

A quantitative approach was therefore pursued, which led to the assignment of a probability for each of the five reference groups, allowing more accurate inferences to be drawn. By this quantitative approach, samples were assigned to a single continental ancestry group whenever the probability for that group was greater than 0.70. When the maximum probability for any single continental group was ≤0.70, the sample was assigned to “multiple groups.” In this way, 89 % of samples were assigned to a single continental or subcontinental group; the remainder were assigned to multiple defined groups. Details of the accuracy of these predictions are given in the following paragraphs.

Out of a total of 1,877 predictions of European ancestry, 93 % were correct as given by self-report/site report of Adygei, Austrian, Azerbaijani, British, Caucasian, Chuvash, European, German, mixed German/Polish, Georgian, Hungarian, Irish, Komi, Polish, or White (meaning likely European). Of the 128 individuals whose predicted European ancestry was not clearly consistent with self-report/site report, 26 were Druze, 24 were Yemenite, 15 were mixed Italian/ Greek/ Syrian, 15 were Uzbeks, 13 were Kazakhstani, 11 were Inuit, 8 were Lebanese, 4 were mixed race, 4 were “Other,” 3 site-reported as African-American, 2 were Iraqi, 1 was Japanese, 1 was Quechua, and 1 was South Asian. Y-chromosome haplogroups were obtained for 52 out of these 128 samples that showed inconsistency between ancestry group inferred from the genome-wide SNPs data and self-/site-recorded ancestry information. Y haplogroups for 49 of these samples are found in people of European paternal origin. Furthermore, the majority of these 128 samples (*N* = 102), including three site-reported African-Americans, had mitochondrial haplogroups which indicated Western Eurasian maternal origin. These Y and mtDNA results, together with the genome-wide results, suggested that despite self-/site-reported ancestry information, many of these 128 individuals share a considerable proportion of their genetic ancestry with Europeans, likely due to recent European admixture. Some may represent cases were continental ancestry is difficult to infer from genetic data because the individuals descended from a geographic area located between major geographic regions (such as from the Middle East or western parts of Asia).

Out of a total of 233 predictions of East Asian ancestry, 94 % were correct as given by self-/site-reported ancestry of Ami, Asian, Atayal, Cambodian, Chinese, Hakka, Japanese, Korean, Laotian, Micronesian, or Yakut. Of the 14 individuals whose predicted East Asian ancestry was not clearly consistent with self-report/site report, one was site-reported as British, two were Gimi, one was Inuit, one was mixed (Italian/Greek/Syrian), one was Kazakh, one was Uzbek, three were Nasioi, and four were Other. The mitochondrial haplogroups for these individuals, except the Gimi and the Nasioi, indicated Eastern Eurasian maternal ancestry suggesting, together with the genome-wide results, considerable East Asian admixture. Six Y-chromosome haplogroups were obtained and all indicated East Asian, Southeast Asian, or Oceanic origin. The finding that mitochondrial and Y-chromosome haplogroups correctly assign Oceanic origin in the Gimi and Nasioi may illustrate the limitations in the resolution level of the genome-wide data in terms of separating Oceanians from East Asians using our approach. This is likely because Oceanic samples are missing in the HapMap 3 reference data used as reference for this analysis.

Of the total of 145 predictions of African ancestry, 88 % were correct as given by self-/site-reported ancestry of African, African-American, Barbadian, Ethiopian, Hausa, or Jamaican. Of the 17 individuals whose predicted African ancestry was not clearly consistent with self-report/site report, 15 were site-reported as “British,” 1 site-reported as Other, and 1 site-reported as White. Without exception, all these 17 samples carried African mitochondrial haplogroups indicating, together with the genome-wide results, considerable African admixture in these samples. Furthermore, all of the four males had African Y haplogroups.

Of the total of 107 predictions of South American ancestry, 98 % were correct as given by self-/site-reported ancestry of Karitiana, Mayan, Pima, Quechua, or Ticuna. Both of the individuals whose genome-wide predicted South American ancestry was not clearly consistent with self-/site-reported ancestry and who classified themselves as Other had mitochondrial haplogroups of Eastern Eurasian origins. Both were female, so Y-chromosome haplogroups were not available.

Of the 24 predictions of South Asian ancestry, 96 % were correct as given by self-/site-reported ancestry of Keralite, mixed Indian/Pakistani, or South Asian. The individual, whose genome-wide predicted South Asian ancestry was not consistent with self-report/site report, was site-reported as British and carried a Western Eurasian mitochondrial haplogroup. This individual was female, so a Y-chromosome haplogroup was not available.

**Fig. 2:**
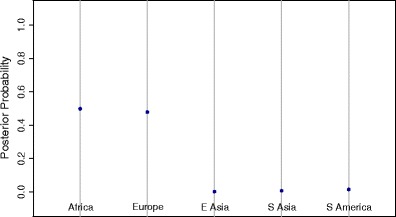
Quantitative assessment of biogeographic ancestry from an individual whose father was of African origin and whose mother was of European origin

Our approach was insightful also in terms of genetically classifying individuals of mixed continental ancestry. Unfortunately, only a handful of such individuals were unblinded with details of their parental origin. Taking a single example, Fig. 2 shows the quantitative assessment of an individual whose father was of African descent and whose mother was of European descent according to the record information. The almost equal proportions of African and European DNA were accurately captured by the method. Furthermore, the determination of the Y-chromosome haplogroup as E-U290, which is observed in Africa, Western Asia, and Europe, and the mitochondrial haplogroup HV, which is observed in Western Eurasia, indicated that the paternal line is African while the maternal line is European, in agreement with record-based ancestry information.

**Fig. 3:**
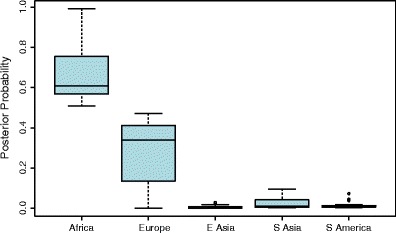
Box plot for quantitative assessments of biogeographic ancestry for 24 Ethiopian individuals

**Fig. 4:**
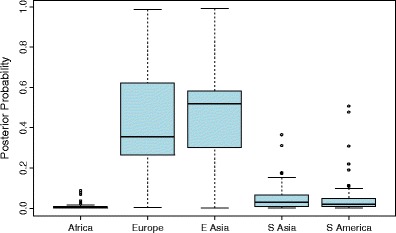
Box plot for quantitative assessments of biogeographic ancestry for 57 individuals from Kazakhstan

Further useful insights were obtained. Figure 3 shows the box plot for 24 individuals of Ethiopian descent. There is a substantial European component compared with the mainly West African reference samples, indicating the genetically admixed situation of North Africans. Similarly, Fig. 4 shows the box plot for 57 individuals from Kazakhstan, which lies on the silk route between China and Europe. It can be seen that individuals who come from a location that is intermediate between the origins of the reference groups may be indistinguishable from an individual of mixed-race origin, using these first-pass techniques. The ongoing development of methods to estimate, for example, LD block size in individuals of mixed ancestry may elucidate the matter further.

#### Inference of eye and hair color categories

Table 1 shows the breakdown of chip-predicted versus site-reported eye color for samples which passed platform quality checks and had both site-reported eye color, as well as a complete genotype profile for the six SNPs required (*N* = 1,136). It can be seen that 70 % of predictions of blue eyes and 85 % of predictions of brown eyes agreed with site-reported eye color using the *p* > 0.7 threshold recommended previously [50].

**Table 1:** Chip-predicted versus site-reported eye color for 1,136 individuals with chip and record information available

Predicted eye color^a^	Site-reported eye color			Total	Accuracy overall	Accuracy *p* > 0.7 threshold
	Blue (%)^b^	Intermediate (%)^c^	Brown (%)^d^			
Blue	428 (63)	205 (30)	50 (7)	683	63 %	70 %
Intermediate	4 (29)	7 (50)	3 (21)	14	50 %	0
Brown	21 (5)	105 (24)	313 (71)	439	71 %	85 %

^a^Using the prediction model described elsewhere [49, 50]

^b^Includes blue, blue-gray, and gray,

^c^Includes heterochromia, blue-green, green, green-hazel, gray-green, yellow, and intermediate

^d^Includes hazel and brown

Table 2 shows the breakdown of predicted versus site-reported hair color for samples which passed platform quality checks and had both site-reported hair color as well as the complete genotype profile of the 18 SNPs required (*N* = 1,137). It can be seen that using the previously developed prediction guide [52], 58 % of predictions of black/dark brown hair corresponded to site report of black, dark brown, or brown hair; 72 % of predictions of brown/light brown/dark blonde corresponded to site report of dark brown, brown, light brown, or dark blonde hair; 63 % of predictions of blonde/dark blonde corresponded to site report of light brown, dark blonde, or blonde hair; and 48 % of predictions of red hair corresponded to site report of red hair.

**Table 2:** Chip-predicted versus site-reported hair color for 1,137 individuals with chip and record information available

Predicted hair color^a^	Site-reported Hair Color					Total
	Black (%)	Dark brown/brown (%)	Light brown/dark blonde (%)	Blonde (%)	Red (%)	
Black/dark brown	70 (10)	351 (48)	138 (19)	41 (6)	133 (18)	733
Brown/light brown	3 (3)	44 (39)	37 (33)	13 (12)	15 (13)	112
Blonde/dark blonde	6 (3)	50 (23)	76 (35)	61 (28)	26 (12)	219
Red	1 (1)	17 (23)	14 (19)	6 (8)	35 (48)	73

^a^Using the prediction model and the prediction guide described elsewhere [52]

#### Inference of relatedness

The proportion of the genome shared IBS was estimated for all pair-wise combinations of the 3,034 samples, using 192,576 markers. For the majority of the samples however, the true relationships were not known/site-reported. The true relatedness of samples was available from one source, for which 3,240 pair-wise comparisons had been performed for 81 samples. In this set, all 27 first-degree relative pairs, ten second-degree relative pairs (four uncles/aunts, five grandparents, and one half-sibling), three third-degree relative pairs (two first cousin pairs and one great aunt), and 3,199 unrelated pairs were correctly identified. One additional pair of individuals was observed to share 9 % of the genome IBS, in line with a fourth-degree relationship (e.g., first cousin once removed). Site report for the two was unrelated, but both were of Caribbean origin. It is acknowledged that the prediction of fourth-degree relationships is only valid in highly outbred populations. For populations which are, or have been in the past, genetically isolated, there will be an overprediction of distant relatives.

## Discussion

The Identitas v1 Forensic Chip is the first all-in-one diagnostic tool targeted for DNA intelligence purposes, allowing for massively parallel genome-wide inference of ancestry, appearance, relatedness, and sex. This DNA chip, manufactured by Illumina using their well-established Infinium technology, is able to deliver highly reproducible genotypes as indicated by the >99.999 % genotyping agreement achieved in an independent comparison with the Infinium 610-Q (Illumina) GWAS array. v1 of the Identitas Forensic Chip exhibited, from samples that passed the quality control threshold of >90 % overall genotype call rate, high predictive power for inference of sex, continental biogeographic ancestry, individual relatedness up to third-degree level, and somewhat less power also for eye and hair color. Even with <90 % call rate, high predictive assignment success was achieved for continental ancestry, although the number of incorrect inferences increases with decreasing overall call rate.

The power of prediction of continental biogeographic ancestry may be explained by the large number of array SNPs used for inference, and the partial redundancy of ancestry information across such SNPs. This also was true for the chip-based inference of relatedness, which was based on an even larger number of SNPs, and for the sex-inference, based on >5,000 X-chromosomal SNPs. The situation is very different for chip-based eye and hair color prediction, where only a small number of particular nonredundant SNPs with high predictive value were used, i.e., six for eye color prediction and 18 for hair color prediction. As long as these particular SNPs are genotyped correctly, eye and hair color prediction can be achieved. The overall call rate therefore provides only a first indication of the chip’s practical performance on ancestry, relatedness, sex, and eye/hair color prediction but should not be viewed as categorical go/no go criteria.

Developing a detailed knowledge about the direct and ideal relationship between DNA quantity and quality, chip performance, and the accuracy of ancestry, relationship and eye/hair color inference requires additional tailored datasets to be generated in the future. One technical complication in the current study was the limited availability of PCR-based sample quantification prior to genotyping. For the majority of samples, PicoGreen provided the only measure of DNA concentration available. Although PicoGreen is known to be reliable in measuring higher DNA concentrations, i.e., tens of nanograms per microliter and beyond, it tends to be less accurate in the single nanogram and sub-nanogram per microliter range encountered in many forensic applications. Similarly, the tolerance of the Identitas v1 Forensic Chip in using degraded DNA for successful inference of ancestry, appearance, and relatedness needs to be followed up with more extensive testing. Based on our preliminary data, the chip genotyping of partially degraded DNA still allowed high predictive value for biogeographic ancestry, but more data are needed to develop clear degradation thresholds. As with DNA quantity, the large number of SNPs used for ancestry, relatedness, and (less so) inference of sex worked in favor of the chip when dealing with degraded DNA.

One challenge in conducting our analyses was the consolidation of data from multiple sources, each with different conventions for recording biogeographic ancestry information. The large geographic categories described here are necessarily simplifications and the accuracy estimates are likely to be conservative. For instance, a total of 682 individuals were British by self-report/site report and it was clear, upon examination of haplogroups, that this term was, in some instances, intended in the sense of “nationality” rather than biogeographic origin. Our genome-wide analysis however grouped them with individuals of true European origin which consequently lowered the accuracy rate obtained for European ancestry assignment. Other simplifications included the categorization of Mayans with South Americans, as well as the categorization of Micronesians with East Asians. The motivation in providing such wide groupings was to maximize the use of the available data.

The estimates of prediction accuracy for eye and hair color obtained here were lower than those previously reported with the IrisPlex system for eye color [49, 50] and the HIrisPlex system for hair color prediction [52]. Whereas all six IrisPlex SNPs together with the IrisPlex eye color prediction model [49, 50] were applied here without modification, only 18 of the previously reported 22 HIrisPlex DNA variants for hair color prediction [48, 52] could be implemented in the Identitas v1 Forensic Chip. An adjusted hair color prediction model was therefore used. The four DNA variants missing on the chip lay in the *MC1R* gene and are known to be predictive of both red hair and dark hair [48, 52]. Indeed, nearly 60 % of individuals with site-reported red hair were missed. This strongly contrasts with only 14 % instances of red hair missed using the HIrisPlex system containing all 22 DNA variants [52]. The mis-categorization of those individuals with red hair had a knock-on effect on the accuracy of other predicted hair color categories. Secondly, in contrast to the previous eye and hair color prediction studies that solely or mainly used single grader color classifications [48–50, 52], the current study used self-reported eye and hair color. The inevitable subjectivity of self-report means that the estimates achieved here are likely to be conservative. For instance, in two different European studies where single-grader eye color phenotyping was applied, the intermediate eye color category was observed at frequencies of 9.6 % [43] and 14 % [50]. These values are considerably lower than the 35 % of self-reported eye colors that fall into the intermediate category from the present study. Previous studies demonstrated that the intermediate eye color category with the six IrisPlex SNPs used here cannot be predicted with as high accuracy as blue and brown eyes [43, 49, 50]. The inflation of the intermediate eye color category is caused by self-reporting errors and also has an impact on the estimated prediction accuracies for blue and brown. Notably, if we exclude the self-reported intermediate eye color individuals from the prediction analysis, we receive much higher accuracies at 90 % for blue and 94 % for brown eye color. These values are similar to the 94 % accuracy for blue and brown achieved in the previous study using single-grader eye color phenotypes [50].

The greatest value of the Identitas v1 Forensic Chip relative to other tools for FDP or tools for other aspects of DNA intelligence is that the various targets for ancestry, appearance, relatedness, and sex are combined in a single all-in-one diagnostic tool. In criminal investigations, in the absence of a match with a reference sample, such insights can dramatically focus the downstream investigations. The DNA-based investigative intelligence obtained can be used in conjunction with, or in the absence of human eyewitness information, to potentially lead to the identification of suspects. The highly accurate inference of relatedness opens further avenues of application, including paternity/relationship resolution, homeland security matters, and the resolution of missing person investigations, through the analysis of found human remains, including in cases of mass disasters. All usage of this (and similar) technology must, of course, comply with the legal requirements of the country in which it is aimed to be applied to practical forensic case work. The technology introduced here therefore offers a novel opportunity of actionable data in a variety of no-match scenarios. In addition, this comprehensive chip requires the consumption of only one aliquot of DNA evidence material. The proposed tool will be a valuable complement to crime-scene short tandem repeat analysis which represents the industry standard for DNA-based identification through direct matching.

Further developments are underway. The current Version 1 of the Identitas Forensic Chip already contains SNPs associated with freckles, moles, curly hair, skin color, earlobe shape, and body height. However, the phenotype prediction values of the currently known markers for these EVCs are not high enough to be practically useful: more predictive DNA markers need to be identified. Subsequent versions of the Identitas Forensic Chip will include additional markers for these and other appearance traits, as they are identified. For instance, the first GWAS studies on facial shape features have just appeared in the literature [66, 67]. Future improvements may also include refining eye and hair color prediction, especially for the more intermediate colors and eventually moving from the current categorical approach to the prediction of continuous shades of eye and hair color.

Many of the geographically diverse samples that were used in the current study can now serve as reference datasets for any future analysis of biogeographic ancestry using the Identitas v1 Forensic Chip. Although not assessed here, the v1 chip already contains SNPs that carry subregional ancestry information such as for Europe, and downstream efforts will include subregional ancestry into the prediction pipeline. This chip and future iterations will be a valuable addition to the forensic geneticist’s toolkit.

## Electronic supplementary material

Below is the link to the electronic supplementary material.

ESM 1 Figure S1 Bar plot of mitochondrial haplogroup origin for individuals who, by site-report, are of African descent (EPS 6 kb)

High Resolution image (JPEG 103 kb)

ESM 2 Figure S2 Bar plot of Y-chromosomal haplogroup origin for males who, by site-report, are of African descent (EPS 7 kb)

High Resolution image (JPEG 125 kb)
