# Supplementary figures and images for: First all-in-one diagnostic tool for DNA intelligence: genome-wide inference of biogeographic ancestry, appearance, relatedness, and sex with the Identitas v1 Forensic Chip
Source: Int J Legal Med. 2012 Nov 13;127(3):559–72. doi: 10.1007/s00414-012-0788-1 (PMC3631519; doi:10.1007/s00414-012-0788-1)

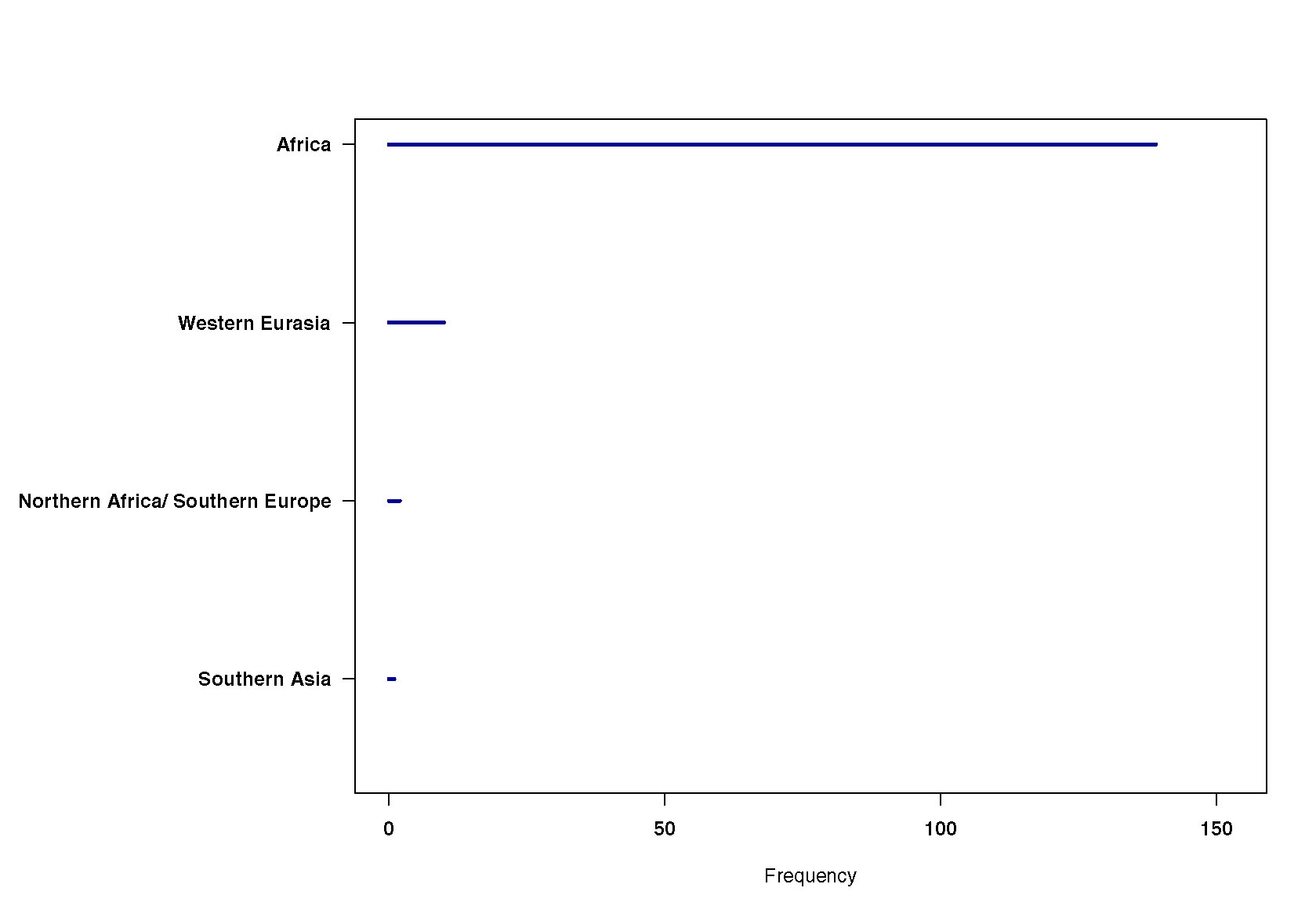

Supplement: Supplementary file 2 — High Resolution image (JPEG 103 kb) [file 414_2012_788_Fig5_ESM.jpg]

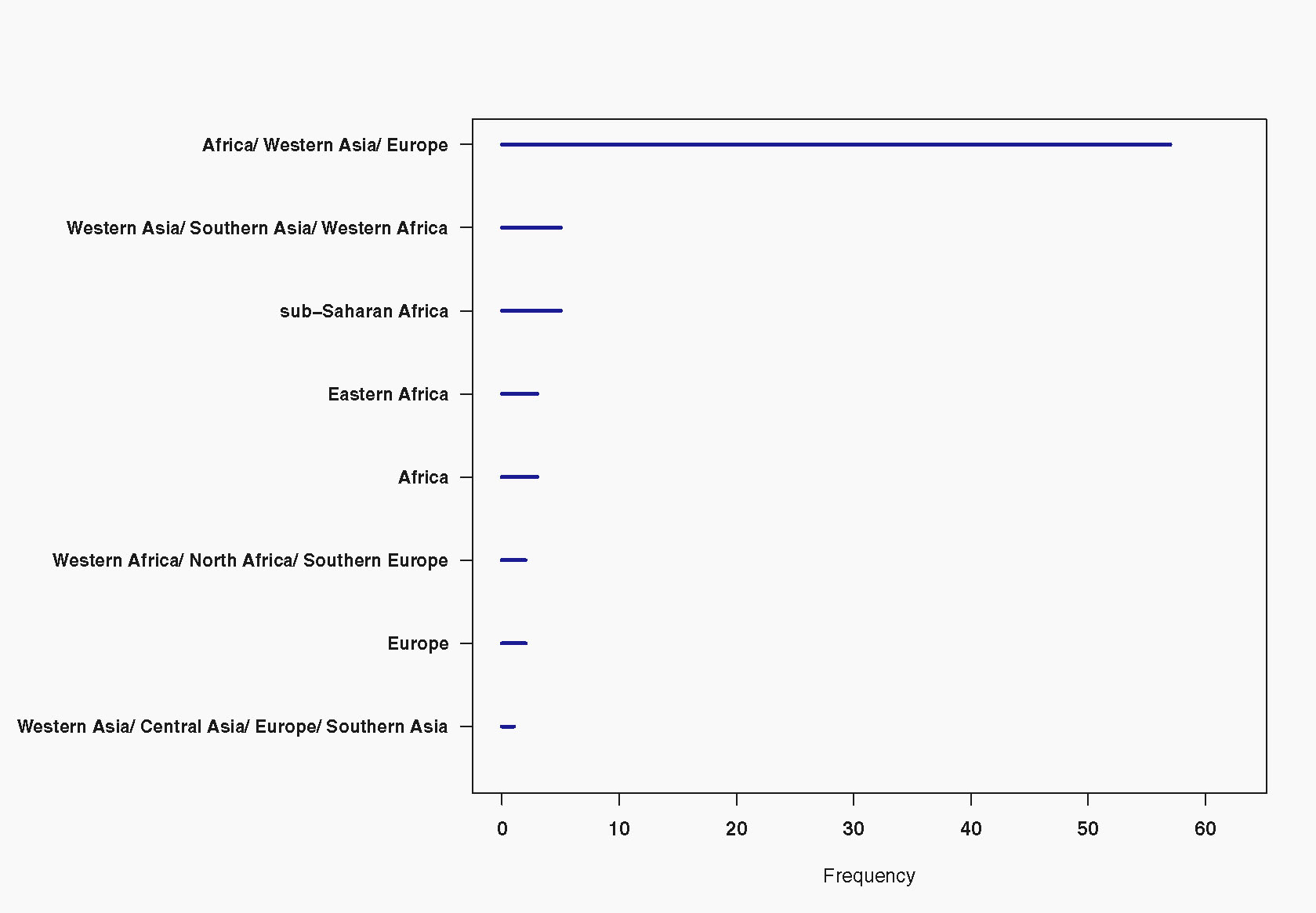

Supplement: Supplementary file 4 — High Resolution image (JPEG 125 kb) [file 414_2012_788_Fig6_ESM.jpg]
